# Influencing factors and risk prediction modeling of maternal postpartum depression: a cross-sectional study in Chinese puerperal women of sitting the month

**DOI:** 10.3389/fpsyt.2023.1252789

**Published:** 2023-09-14

**Authors:** Xiaojuan Su, Yuezhen Zhang, Meide Chen, Huifang Wang, Guihua Liu

**Affiliations:** ^1^Department of Nursing, Quanzhou Medical College, Quanzhou, Fujian, China; ^2^Nursing Department, Quanzhou Women and Children’s Hospital, Quanzhou, Fujian, China; ^3^Department of Obstetrics and Gynecology, Quanzhou Medical College, Quanzhou, Fujian, China; ^4^Department of Child Health Care, Fujian Maternity and Child Health Hospital College of Clinical Medicine for Obstetrics and Gynecology and Pediatrics, Fujian Medical University, Fuzhou, Fujian, China

**Keywords:** sitting the month, postpartum depression, partner support, prediction modeling, puerperal women

## Abstract

**Objective:**

This study aims to investigate the occurrence of maternal postpartum depression (PPD) during menstruation and analyze the influencing factors and risk prediction modeling of maternal PPD in Chinese puerperal women of sitting the month.

**Methods:**

A total of 286 mothers were selected using convenience sampling, who came for a routine postpartum follow-up visit were surveyed, including face-to-face, telephone, and online. They completed questionnaires including the basic profile questionnaire, Postpartum Partner Support Scale (PPSS), Edinburgh PPD Scale (EPDS), Parenting Self-Efficacy Scale (SICS), and Simple Coping Style Questionnaire (SCSQ), who were advised to complete the survey alone, in private, reducing the impact of husband^’^s presence on the quality of the questionnaire. Variables showing statistical significance in the one-way analysis were further analyzed using logistic regression analysis. The predictive value of the logistic regression model was analyzed using the Receiver Operating Characteristic Curve (ROC), and the predictive reliability was expressed as the area under the ROC [Area Under the Curve (AUC)].

**Results:**

The total score of PPD was 7.78 ± 4.57, and 22 people (7.69%) experienced depression during the postpartum period. PPD was found to be correlated with postpartum partner support, positive coping, negative coping, and parenting self-efficacy, with correlation coefficient values of −0.63, 0.62, 0.56, and − 0.70, respectively (all *p* < 0.05). Logistic regression analysis revealed that postpartum partner support and parenting self-efficacy were independent factors influencing PPD, with odds ratios (95% confidence intervals) of 0.76 (0.61 ~ 0.94) and 0.83 (0.75 ~ 0.93), respectively both *p* < 0.05.The area under the curve, sensitivity, and specificity for postpartum partner support and parenting self-efficacy were 1.00 (95% confidence intervals 0.99 ~ 1.00), 99.24, and 90.91%.

**Conclusion:**

Postpartum partner support and parenting self-efficacy independently predict the occurrence of PPD. Healthcare professionals and maternal families should prioritize timely attention to maternal partner support and parenting issues to reduce the occurrence of PPD.

## 1. Introduction

Postpartum depression (PPD) is a debilitating condition characterized by a spectrum of symptoms, including maternal anxiety, depression, insomnia, irritability, sadness, loneliness, cognitive deficits, diminished self-esteem, inadequate coping skills, loss of interest in activities, sleep disturbances, and even severe cases of suicidal thoughts, impacting mothers (1, 2). The prevalence of PPD varies significantly across countries and regions. This paper presented a systematic review and meta-analysis of 58 studies encompassing 37,294 women, aiming to provide a comprehensive overview of PPD incidence. Findings indicated an overall incidence of 17% (3) among healthy mothers with no history of prior depression. Notably, the Middle East exhibited the highest prevalence (26%), while Europe reported the lowest (8%). Another systematic review and meta-analysis revealed a comparable prevalence of 14% (4). Developing countries displayed a markedly elevated rate of PPD, with China^’^s prevalence notably exceeding the global average at 21.4%.

Postpartum depression presents a substantial risk to maternal well-being, infant health, and overall family dynamics (5). Despite being a commonly occurring condition, PPD frequently remains undetected and unaddressed (6), resulting in various detrimental consequences. Undiagnosed, untreated or insufficiently treated depression can yield deleterious long-term outcomes. In the case of mothers, PPD is linked to suboptimal physical and psychological well-being, culminating in diminished quality of life and potential psychiatric institutionalization. Moreover, PPD can detrimentally influence a child’s cognitive, social, and socioemotional progress, resulting in reduced intelligence quotient, behavioral challenges, and perturbing family dynamics (7–10).

The etiology of PPD remains elusive, nevertheless, it is risk and mitigation factors encompass psychological, biological, social, and cultural dimensions. A recent systematic review and meta-analysis highlighted a prominent concentration of risk factors within the realm of insufficient social support (11), encompassing spousal, peer, and healthcare professional support. In the realm of Chinese culture, the postpartum phase frequently encompasses a customary practice referred to as “sitting the month,” which prioritizes the provision of exclusive care for newborns and rest for mothers during the initial month following childbirth. This confinement period may limit timely support from relatives and friends, thereby increasing reliance on spousal support. The conducted research has unveiled noteworthy correlations between the presence of strained relationships with spouses or family members and the incidence of PPD among Chinese women (12, 13). Previous research had indicated a substantial inverse association between partner support during this period and PPD (14, 15). In the aftermath of childbirth, women assume the responsibility of infant care, wherein perceived parenting satisfaction and self-efficacy play vital roles. The association between parenting self-efficacy and PPD was supported by a comprehensive meta-analysis of 114 cohort studies. This analysis indicates that the stress resulting from the changing roles and responsibilities of parenthood can be considered a significant risk factor for PPD (16). A longitudinal study conducted over a span of 4 years in the United Kingdom unveiled a noteworthy association between reduced parental self-efficacy and depression within a demanding parenting context (17). Additionally, the categorization of coping styles into positive and negative facets underscores the predictive potential of negative coping strategies for PPD (18).

Nevertheless, the relationship between maternal PPD and partner support during the traditional practice of “sitting the month” has yet to be determined. The unique cultural context of this period may impact women’s social support systems, with husbands playing a pivotal role. Given the observed negative correlation between partner support and PPD, the lack of a quantitative measurement tool for postnatal partner support in China necessitated the validation of the quantifiable postnatal partner support scale. Most studies (2–4) solely focused on describing the epidemic characteristics, without application of Simple Coping Style Questionnaire or providing prediction model for postpartum partner support and parenting self-efficacy.

In response to this gap in scholarship, we aim to employ this validated scale, along with the Postpartum Partner Support Scale, Self-efficacy in Infant Care Scale, and Simple Coping Style Questionnaire, identify the influencing factors of PPD and establish a targeted early warning model to create effective tools for helping spotting PPD. We also aim to assist healthcare professionals in early identification and targeted prevention of PPD, thus addressing a critical gap in the literature.

## 2. Methods

### 2.1. Participants and procedure

#### 2.1.1. Sampling and sample size

In this study, we employ a simple convenience sampling approach to investigate the prevalence of PPD. Utilizing qualitative data, specifically an overall rate estimation, we apply the formula to determine the required sample size (19).


$$n={\left(Z\times \delta \right)}^{2}p\left(1-p\right)$$

Here, n signifies the sample size, Z denotes the Z-statistic corresponding to a confidence level (at 95% confidence, Z = 1.96), δ represents the allowable absolute error, typically set at 0.05 for acceptable precision, and *p* signifies the overall rate. With existing literature reporting a 17% prevalence of PPD via meta-analysis, corroborated by a substantial body of research (3), our study utilizes p = 0.17 to calculate a minimum sample size of n = 216. To account for potential nonresponse and variations in maternal conditions, a total of 300 questionnaires were distributed in this study, with 286 valid questionnaires returned, yielding an effective rate of 95.33%.

#### 2.1.2. Ethical considerations

Our study received ethical clearance from the Ethics Committee of Quanzhou Medical College, Quanzhou, Fujian, China (Ethics Code: No. 2021007). The central tenet of voluntary participation was upheld throughout the study. Prior to survey administration, all participants were thoroughly briefed on the study’s objectives, methodologies, potential benefits, and risks. Informed consent was meticulously obtained from every participant, establishing a foundation of trust and transparency.

#### 2.1.3. Participant privacy

In accordance with ethical guidelines, a range of strategies were deployed for survey completion. Face-to-face participants were afforded privacy during survey completion, ensuring responses were provided independently and without influence. Similarly, participants completing surveys via telephone or online platforms were instructed to do so in a private setting. The preservation of participant anonymity was ensured through sealed envelopes, preventing any inadvertent bias during data collection. Our rigorous approach aimed to mitigate potential stressors and maintain the integrity of collected data.

#### 2.1.4. Study design and participants

A convenience sampling method was employed to select women who had given birth between December 2021 and September 2022 at a hospital of Quanzhou Women and Children’s Hospital in Quanzhou City as the study population. The inclusion criteria were as follows: (1) women in the postpartum period (within 28 days after delivery) and (2) those with clear consciousness and informed consent to participate. Exclusion criteria included: (1) unwillingness or non-cooperation with the survey; (2) visual or hearing impairment that hindered understanding and response to the questionnaire; and (3) those who reported a history of depression or psychiatric disorders before or during pregnancy.

Study participants were recruited at the hospital where they delivered. After delivery, mothers provided their community address, detailed home address, personal phone numbers, and their husband’s contact information to facilitate postpartum health education and follow-up visits.

Medical professionals, including doctors, midwives, nurses, and researchers from the study group, were responsible for recruiting study subjects. Before conducting the survey, the researcher in charge of recruiting subjects received comprehensive training, including an understanding of the questionnaire items and survey guidelines. Precautions, such as maintaining subject confidentiality and ensuring that participants completed the questionnaire independently, were emphasized.

#### 2.1.5. Challenges in participant recruitment

Conducting research during the novel coronavirus pandemic presented challenges in reaching potential participants. Lockdowns, movement restrictions, and concerns about hospital visits impacted the availability of eligible mothers. We coordinated closely with the hospital administration and medical staff to ensure that the recruitment process followed safety protocols. In cases where physical presence was challenging, we incorporated telephonic and online methods for data collection. By providing flexible options, we aimed to accommodate participants’ preferences and alleviate concerns related to the pandemic.

The cultural norm of “sitting the month” during postpartum limited women’s mobility and access to support networks, potentially affecting their willingness to participate in the study. We provided comprehensive explanations to potential participants about the nature of the research, assuring them of confidentiality and the importance of their contribution to maternal health research.

The postpartum period is characterized by significant time demands, making it challenging for new mothers to allocate time for research participation. We designed the survey instruments to be concise and user-friendly, minimizing the time required for completion. Moreover, we offered multiple modes of participation (face-to-face, telephone, and online), allowing participants to choose the most convenient option that aligned with their schedules.

### 2.2. Measurement

#### 2.2.1. General demographic information questionnaire

This questionnaire collected maternal and husband^’^s age, education, gestational age of the newborn at birth, sex of the newborn, type of delivery, the number of pregnancies, the number of deliveries, the gestational weeks, maternal education level, family income, the sex of the babies, feeding method, and mother-in-law/daughter-in-law relationship.

#### 2.2.2. Postpartum partner support scale

The PPSS comprises 20 items, scored on a scale of 1–4, representing strongly disagree to strongly agree. The total score ranges from 20 to 80, with a higher score indicating a higher level of postpartum partner support. The English version demonstrated a Cronbach’s alpha coefficient of 0.96, a goodness-of-fit index CFI of 0.99, and a root mean square error of approximation SRMR of 0.05, indicating good model fit (14). To ensure the reliability of the scale, a Chinese version was developed and tested by our research group. The Chinese version exhibited a Cronbach’s alpha coefficient of 0.968 and a retest reliability coefficient of 0.905 (*p* < 0.01). In the validation factor analysis, the χ^2^/df was 1.476, and the RMSEA was 0.047.

#### 2.2.3. Edinburgh postpartum depression scale

The EPDS consists of 10 items, with each item scored from 0 to 3. The total score ranges from 0 to 30, with higher scores indicating greater severity of maternal depression. A total score of ≥13 is considered indicative of PPD, with a significant increase in specificity of 0.95 (20). EPDS was commonly used to assess the degree of PPD, and many studies reported good validity and reliability of the Edinburgh tool in different contexts, from 0.76 to 0.861 (21, 22), and The Cronbach’s alpha of EPDS in this study was 0.853.

#### 2.2.4. Self-efficacy in infant care scale

The SICS consists of 44 items, each item indicates one parenting task; the higher the score, the higher the self-efficacy. For instance, women were asked to rate their degree of confidence in performing designated tasks/situations; a score ranging from 0 to 100 was assigned, depending on their confidence response from “not confident at all to do it” to “definitely confident I can do it.” The scale is scored by summing the numerical ratings for each task and dividing the result by the number of tasks. The reported internal consistency was 0.94 for the total scale (23). The Cronbach’s alpha of SICS in this study was 0.950.

#### 2.2.5. Simplified coping style questionnaire

The SCSQ was used to measure the coping style of Chinese puerperal mothers，comprised to two dimensions, positive coping and negative coping, with a total of 20 items. Each item is scored from 0 to 3. A high score of the positive coping indicates that the individuals are more inclined to adopt positive coping styles with efforts to solve a stressful situation/problem, and a high score of the negative coping refers that the individuals are more inclined to adopt negative coping styles with distancing or avoiding a stressful situation. The Cronbach’s alpha coefficient of the positive coping and negative coping was 0.863 and 0.741 (24). The Cronbach’s alpha of SCSQ in this study was 0.897 and 0.862.

### 2.3. Data collection methods

Medical professionals, including doctors, midwives, nurses, and researchers from the study group, were responsible for recruiting study subjects. Before conducting the survey, the researcher in charge of recruiting subjects received comprehensive training, including an understanding of the questionnaire items and survey guidelines. Precautions, such as maintaining subject confidentiality and ensuring that participants completed the questionnaire independently, were emphasized.

Study participants were recruited at the hospital where they delivered. After delivery, mothers provided their community address, detailed home address, personal phone numbers, and their husband’s contact information to facilitate postpartum health education and follow-up visits. Data collection was carried out through face-to-face interviews (using pen and printed paper questionnaires), telephone interviews, and online questionnaires. In face-to-face interviews, the survey was conducted in a private room with only the mother and newborn present. For telephone or online surveys, mothers were informed that the questionnaire would evaluate their husband’s support and were requested to ensure their husband temporarily left during the survey to allow the wife to complete the questionnaire independently.

### 2.4. Statistical analysis

Data collected were entered using Epidata 3.0, and descriptive statistical analysis, Pearson’s correlation analysis, and logistic regression analysis were performed using SPSS 22.0. General demographic data were analyzed using percentages and frequencies. Postpartum husband support, parenting self-efficacy, coping style, and PPD were described using mean ± standard deviation and median (interquartile spacing). Pearson’s correlation analysis was employed to examine the correlation between PPD and postpartum husband support, as well as parenting self-efficacy and coping style. We used the Shapiro–Wilk method to group the data (depressed and non-depressed groups) to check if the data conformed to a normal distribution. If the variable was normally distributed, an independent *t*-test was used to compare PPD according to two groups. If the variable was not normally distributed, the Mann-Whitney U test was used to compare PPD according to two groups. Variables showing statistical significance in the one-way analysis were further analyzed using logistic regression analysis. The predictive value of the logistic regression model was analyzed using the Receiver Operating Characteristic Curve (ROC), and the predictive reliability was expressed as the area under the ROC [Area Under the Curve (AUC)]. Hypothesis testing employed a two-sided test with a significance level of 0.05. A value of *p* less than 0.05 indicated statistically significant differences.

## 3. Results

### 3.1. General information

The mean age of the mothers was 29.83 ± 3.65 years, ranging from 17 to 39 years. The mean age of the husbands was 30.43 ± 3.75 years, ranging from 22 to 42 years. Among the participants, 78 (27.3%) had completed high school or junior college, 112 (39.2%) had a bachelor’s degree or above, and 2 (0.7%) had an elementary school education or below. In terms of maternal personality type, 146 (51.0%) were introverts and 140 (49.0%) were extroverts. The number of pregnancies ranged from 1 to 4, with an average of 2.04 ± 0.82, and the number of deliveries ranged from 0 to 3, with an average of 1.62 ± 0.70. Among the participants, 210 (73.4%) had normal deliveries and 76 (26.6%) had cesarean deliveries. The gestational weeks ranged from 34^+5^ to 41^+0^. The sex of the babies was 160 (55.9%) male and 126 (44.1%) female. Regarding feeding method, 120 (42.0%) exclusively breastfed, 52 (18.2%) used formula feeding, and 114 (39.9%) used mixed feeding. Among relationship of mother-in-law/daughter-in-law, 62 (21.7%) think it is not good, 138 (48.3%) think it is general, and 86 (30.1%) think it is good.

### 3.2. Incidence of PPD and scores of EPDS, PPSS, SICS, and SCSQ

The EPDS scores of the 286 women during the menstrual period ranged from 0 to 27, with a mean score of 7.78 ± 4.57. A total score of ≥13 was considered indicative of PPD, with 22 participants meeting this criterion, resulting in a positive rate of 7.69%. The PPSS scores ranged from 20 to 80, with a mean score of 58.20 ± 16.29. The SICS total mean scores ranged from 29.32 to 100, with a mean score of 71.70 ± 14.89. The positive coping scores ranged from 1 to 36, with a mean of 23.25 ± 6.82, and the negative coping scores ranged from 1 to 24, with a mean of 9.75 ± 5.43.

### 3.3. Correlation analysis results

Pearson correlation analysis revealed significant correlations between the total PPD score and the total partner support, positive coping, negative coping, and parenting self-efficacy scores. The correlation coefficient values were − 0.63 (*p* < 0.001), 0.62 (*p* < 0.001), 0.56 (*p* < 0.001), and − 0.70 (*p* < 0.001), respectively, all of which were statistically significant.

### 3.4. Univariate analysis results of factors influencing PPD

Univariate analysis showed that postpartum partner support, coping style, parenting self-efficacy score, maternal personality type, mode of delivery, and mother-in-law-daughter-in-law relationship significantly influenced PPD (*p* < 0.05). However, factors such as maternal and husband age and education, number of weeks of pregnancy, infant gender, expected baby’s sex, feeding method, and family economy did not have a significant effect on PPD (*p* > 0.05). Detailed information can be found in Table 1.

**Table 1:** Univariate analysis of factors influencing PPD.

	Projects	Number of cases (*n*)	PPD group a *n* (%) or ($\bar{X}$ ±s)	Non-PPD group b *n* (%) or ($\bar{X}$±s)	*x*^2^/Z	*p*
	Total partner support score	286	26.5 (10.25)	63 (12.75)	−7.62	<0.001
	Positive response to total score	286	9 (9.75)	25 (8)	−6.68	<0.001
	Total negative response score	286	21 (4)	8 (7)	−7.13	<0.001
	Total parenting self-efficacy score	286	39.20 (10.28)	76.14 (19.09)	−7.48	<0.001
	Maternal age	286	28 (3)	30 (6)	−1.94	0.05
	Husband’s age	286	30 (3)	30 (5)	−1.55	0.12
	Number of pregnancies	286	2 (1)	2 (2)	−1.86	0.06
	Number of deliveries	286	1 (1)	1.5 (1)	−1.60	0.11
	Number of days after delivery	286	18 (4)	19 (9)	−0.99	0.32
Maternal education	Elementary school and below	2	0 (0.0)	2 (0.8)	5.90	0.16
	Junior High School	36	0 (0.0)	36 (13.6)		
	High School and Junior College	78	4 (18.2)	74 (28.0)		
	College	58	6 (27.3)	52 (19.7)		
	Bachelor’s degree and above	112	12 (54.5)	100 (37.9)		
Husband’s education	Elementary school and below	2	0 (0.0)	2 (0.8)	8.23	0.06
	Junior High School	40	0 (0.0)	40 (15.2)		
	High School and Junior College	58	2 (9.1)	56 (21.2)		
	College	78	10 (45.5)	68 (25.8)		
	Bachelor’s degree and above	108	10 (45.5)	98 (37.1)		
Personality Type	introverted	146	16 (72.7)	130 (49.2)	4.48	0.04
	Extrovert	140	6 (27.3)	134 (50.8)		
Household Economy	Difference	36	4 (18.2)	32 (12.1)	0.94	0.64
	General	246	18 (81.8)	228 (86.4)		
	Good	4	0 (0.0)	4 (1.5)		
Delivery method	easy childbirth	210	12 (54.5)	198 (75.0)	4.36	0.04
	Cesarean delivery	76	10 (45.5)	66 (25.0)		
Baby Gender	Male	160	12 (54.5)	148 (56.1)	0.02	1.00
	Female	126	10 (45.5)	116 (43.9)		
Expected baby gender	Male	114	12 (54.5)	102 (38.6)	3.22	0.25
	Female	20	0 (0.0)	20 (7.6)		
	Let nature take its course	152	10 (45.5)	142 (53.8)		
Feeding method	Exclusive breast milk	120	6 (27.3)	114 (43.2)	2.50	0.27
	Artificial feeding	52	6 (27.3)	46 (17.4)		
	Mixed feeding	114	10 (45.5)	104 (39.4)		
Mother-in-law and daughter-in-law relationship	Not good	62	12 (54.5)	50 (18.8)	18.94	<0.001
	General	138	10 (45.5)	128 (48.4)		
	Very good	86	0 (0.0)	86 (32.5)		

^a^*n* = 22, ^b^ *n* = 264; PPD, Postpartum depression; Z, Mann Whitney u test.

### 3.5. Logistic regression analysis results of factors influencing PPD

Logistic regression analysis was performed with the occurrence of PPD as the dependent variable and the eight factors that showed significant differences in the univariate analysis (partner support, positive coping, negative coping, parenting self-efficacy score, maternal personality type, mode of delivery, and mother-in-law-daughter-in-law relationship) as independent variables. Logistic regression analysis revealed that postpartum partner support and parenting self-efficacy were independent factors influencing PPD, with odds ratios (95% confidence intervals) of 0.76 (0.61 ~ 0.94) and 0.83 (0.75 ~ 0.93), respectively, both *p* < 0.05. Further details are provided in Table 2.

**Table 2:** Logistic regression analysis of factors influencing PPD.

Influencing factors	*B*	*S.E.*	*Wals* *x*^2^	OR	95% CI	*p*
Constants	17.74	4.63	14.69			
Postnatal partner support	−0.28	0.11	6.22	0.76	0.61 ~ 0.94	0.01
Parenting self-efficacy	−0.19	0.06	11.30	0.83	0.75 ~ 0.93	0.00

PPD, Postpartum depression; 95% CI, 95% Confidence interval.

### 3.6. ROC analysis of postpartum partner support and parenting self-efficacy to predict PPD

**Figure 1:**
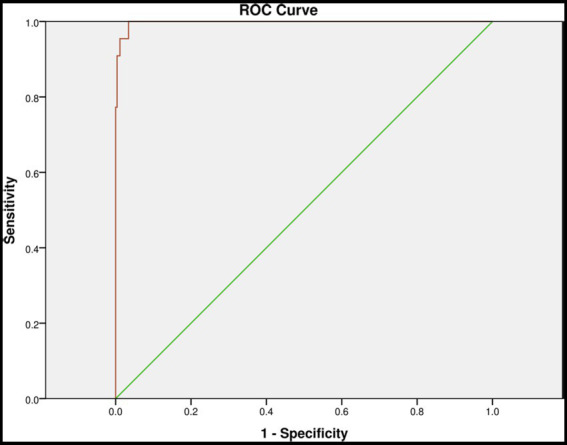
Receiver Operating Characteristic Curve (ROC) curves for postpartum partner support and parenting self-efficacy in assessing the occurrence of PPD.

The area of the curve for postpartum partner support and parenting self-efficacy analyzed by ROC curve was were 1.00 (95% confidence intervals 0.99–1.00), *p* < 0.001. The sensitivity and specificity for postpartum partner support and parenting self-efficacy were 99.24 and 90.91%. For details, see Table 3 and Figure 1.

**Table 3:** ROC curves for postpartum partner support and parenting self-efficacy in assessing the occurrence of PPD.

Area	Std. Error^a^	Asymptotic Sig.^b^	95% CI
1.00	0.00	<0.001	0.99 ~ 1.00

^a^Under the nonparametric assumption; ^b^Null hypothesis: true area = 0.5; 95% CI, 95% Confidence interval; ROC, Receiver operating characteristic curve.

## 4. Discussion

### 4.1. Analysis of the current situation of PPD

This study examined the present state of PPD during the traditional Chinese practice of “sitting the month” and analyze the factors that influence maternal PPD in Chinese puerperal women. Our study indicated that a threshold of 10 resulted in sensitivity and specificity values of 0.85 and 0.84, respectively, while a threshold of 13 yielded sensitivity and specificity values of 0.66 and 0.95, respectively (20). Given the evolving landscape of PPD research in China, with recent studies adopting an EPDS threshold of 13 for diagnosis (25–27), our study also employs the EPDS threshold of 13 for diagnosing PPD.

Our results showed that 22 (7.69%) of the women were identified as having PPD. This incidence rate is lower than that reported in previous domestic studies (28, 29). The variation in the incidence of PPD across studies may be attributed to different screening thresholds used in the EPDS scale. Chen and Hu et al. used a threshold of EPDS ≥10 to determine PPD, resulting in a higher incidence rate (28, 29). Additionally, the incidence of PPD can vary among different cities due to variations in maternal lifestyle, work, and economic pressure. Researchers discovered a significant incidence of postpartum depression (19.8–31.0%) within the urban region of Beijing (25), Wuhan (28), and Hangzhou (27), which are categorized as a first-tier city. In the current study, the participants were mothers who gave birth in three hospitals in Quanzhou, a second-tier city, which may explain the slightly lower level of PPD observed (29). However, it is important to note that future research should aim to increase the sample size, expand the scope of the survey, enhance sample representativeness, and incorporate qualitative interviews and objective evaluation methods to conduct more comprehensive and accurate assessments of the current situation of PPD.

### 4.2. Correlation between postpartum partner support and PPD

In this study, the mean score of postpartum partner support was 58.20 (SD = 16.29), which was slightly lower than the result reported by Eslahi et al. (15) of 64.32 (SD = 10.45). The mean score of the depressed group in this study was 25.73 (SD = 4.81), while the mean score of the non-depressed group was 60.90 (SD = 13.79), which was slightly lower than the result reported by Dennis et al. (14) of 68.09 (SD = 9.52). There was a significant negative correlation between postpartum partner support and PPD (*r* = −0.626, *p* < 0.001), which is consistent with the findings of Dennis (*r* = −0.30, *p* < 0.01) (14) and Eslahi (*r* = −0.39, *p* < 0.001) (15). During the “sitting the month” a traditional Chinese custom where mothers are subjected to various restrictions during the month after delivery, limited socialization and support from family and friends can increase the risk of PPD (30). The spouse is considered the most important source of maternal support during this period. Furthermore, women require support from their partners for infant care, household chores, and emotional well-being (30, 31). Studies had shown that women with poor relationships have at least four times higher incidence of PPD compared to those with good relationships (32).

Therefore, the period of “sitting the month” is a critical time for maternal physical and psychological recovery, and healthcare professionals should acknowledge its importance in postpartum care, especially during the Corona Virus Disease 2019 (COVID-19) pandemic. Maternal partners play a crucial role as a readily accessible source of social support, and postpartum visits should focus on assessing the status of postpartum partner support. Identifying mothers with inadequate postpartum partner support in a timely manner and providing appropriate education and counseling programs for spouses can help reduce the occurrence of PPD and improve the overall well-being of mothers and infants.

### 4.3. Effect of parenting self-efficacy on PPD

Parenting self-efficacy refers to parents’ sense of competence and confidence in their ability to successfully perform various parenting tasks, such as feeding and soothing (33). Our results showed that the total mean score of parenting self-efficacy ranged from 29.32 to 100, with a mean of 71.70 (SD = 14.89). The score of the PPD group of 40.99 (SD = 8.95) was significantly lower than that of the non-depression group of 74.26 (SD = 12.18), and a significant negative correlation (*r* = −0.698, *p* < 0.05) was observed between parenting self-efficacy scores and PPD.

Logistic regression analysis also identified parenting self-efficacy as an influential factor for PPD. Hence, it is imperative for healthcare providers to prioritize not only the physical and psychological well-being of mothers during postpartum care and visits, but also to offer a comprehensive range of parenting information and guidance through various online and offline channels. Enhancing mothers’ knowledge of parenting and providing timely feedback on the health status of newborns can improve their sense of parenting competence and confidence, thus reducing the incidence of PPD.

### 4.4. The predictive effect of the maternal PPD risk model

The ROC curve is an analytical method represented as a graph, which is employed to assess the performance of binary diagnostic classification methods. It has been widely adopted in the field of medicine to evaluate the efficacy of diagnostic approaches (34–38). The AUC is a frequently used metric for gaging the accuracy of diagnostic tests. An ideal ROC curve has an AUC of 1.0. For a diagnostic technique to hold meaning, the AUC must exceed 0.5, and in general, it should surpass 0.8 to be deemed acceptable. The AUC is often accompanied by a 95% confidence interval (CI) because data collected from samples are not fixed values; they are subject to statistical errors. The 95% CI establishes a range of possible values around the true value. Hence, for any test to carry statistical significance, the lower 95% CI value of the AUC must be greater than 0.5 (39).

In this study, the AUC of the maternal PPD risk model constructed during the “sitting the month” period was1.00, well above the 0.8 threshold for acceptability. The 95% CI was (0.99, 1.00), and *p* < 0.001. The sensitivity and specificity were 99.24 and 90.91%. These results suggest that postpartum partner support and parenting self-efficacy can serve as valuable indicators for predicting PPD. Healthcare professionals can employ this early warning model to prevent and promptly identify PPD in women during the “sitting the month” period.

### 4.5. Research limitations and future research directions

Our research employment of convenience sampling introduces potential bias in participant selection. Consequently, our sample may not fully represent the broader population of postpartum mothers experiencing “sitting the month,” may introduce selection bias, challenging the representativeness of our findings. Consequently, the generalizability of our results beyond our specific study population could be restricted.

Recognizing the significance of representative samples, future research endeavors could embrace more rigorous sampling methods, such as random sampling or stratified sampling. By enhancing the representativeness of the sample, the external validity of study results could be bolstered. Additionally, expanding the scope beyond Quanzhou City to include multiple hospitals and diverse regions could provide a more comprehensive insight into the prevalence of postmenstrual depression during “sitting the month.”

In addition to broadening the study’s geographical scope, the temporal span could be extended longitudinally. Beyond the “sitting the month” period and 4 weeks postpartum, future studies could investigate the enduring relationship between maternal depression and partner support. This expanded timeframe would contribute to a more nuanced understanding of the interplay between maternal mental health and partner dynamics.

Future research should aim to expand sample sizes, utilize qualitative interviews, and employ objective evaluation methods to provide a more accurate understanding of the current situation of PPD.

## 5. Conclusion

We have successfully identified the significant protective influence of postpartum partner support and parenting self-efficacy, which exhibit a strong predictive capacity for PPD. Our findings highlight the importance of assessing postpartum partner support and providing appropriate support and education programs during the “sitting the month” to mitigate the risk of PPD. Furthermore, the reduction of PPD can be facilitated by augmenting parenting self-efficacy through comprehensive postpartum care and the provision of parenting information.

## Data availability statement

The original contributions presented in the study are included in the article/supplementary material, further inquiries can be directed to the corresponding author.

## Ethics statement

The studies involving humans were approved by the Ethics Committee of Quanzhou Medical College, Quanzhou, Fujian, China (Ethics Code: No. 2021007). The studies were conducted in accordance with the local legislation and institutional requirements. Written informed consent for participation in this study was provided by the participants’ legal guardians/next of kin. Written informed consent was obtained from the minor(s)’ legal guardian/next of kin for the publication of any potentially identifiable images or data included in this article.

## Author contributions

XS: direct participation, including conceiving and designing the experiment, implementing the study, collecting data, and analyzing and interpreting the data; article writing, including drafting the article and critical review of the intellectual content of the article; and work support, including statistical analysis and obtaining research funding. YZ: direct participation, including conceiving and designing the experiment; article writing, including critical review of the intellectual content of the article; and work support, including mentoring. MC: direct participation, including conceiving and designing the experiment, implementing the study, and collecting data; article writing, including critical review of the intellectual content of the article; and work support, including statistical analysis and mentoring. HW: direct participation, including conceiving and designing the experiment, implementing the study, collecting data, and analyzing and interpreting the data; article writing, including drafting the article; and work support, including statistical analysis. GL: direct participation, including conceiving and designing the experiment, implementing the study, and analyzing and interpreting the data; article writing, including drafting the article and critical review of the intellectual content of the article; and work support, including statistical analysis and mentoring. All authors contributed to the article and approved the submitted version.

## Funding

This research was supported by the fundamental project Guided Science and Technology Plan Project of Quanzhou City (2021N137S) and School-level project of Quanzhou Medical College (XJS2114B).

## Conflict of interest

The authors declare that the research was conducted in the absence of any commercial or financial relationships that could be construed as a potential conflict of interest.

## Publisher’s note

All claims expressed in this article are solely those of the authors and do not necessarily represent those of their affiliated organizations, or those of the publisher, the editors and the reviewers. Any product that may be evaluated in this article, or claim that may be made by its manufacturer, is not guaranteed or endorsed by the publisher.
